# Prevalence And Risk Factors Of Eating Disorders In The Tunisian General Population

**DOI:** 10.1192/j.eurpsy.2024.414

**Published:** 2024-08-27

**Authors:** M. Turki, A. Hadj Ali, G. Chakchouk, S. Ellouze, N. Halouani, J. Aloulou

**Affiliations:** Psychiatric department B, Hedi Chaker University Hospital, Sfax, Tunisia

## Abstract

**Introduction:**

Eating disorders (ED) negatively affect physical, mental, and social well-being. The exact psychopathology of ED is still unknown, with research suggesting the interplay of a combination of factors.

**Objectives:**

The aim of our study was to estimate the prevalence of ED in the Tunisian general population, and to identify associated risk factors.

**Methods:**

We conducted a cross-sectional, descriptive and analytical study among Facebook group members, using an online questionnaire, over the period from February 17, 2023 to May 26, 2023. All respondents over the age of 18 were included in the study. All participants filled a socio-demographic questionnaire. The Eating Attitudes Test (EAT-26) was used to screen for those at risk of eating disorders.

**Results:**

A total of 528 responses were included in the study. 33.3±11.95 years. The subjects were unmarried in 63.4% of cases, of low socio-economic level in 19.5%, with a university education in 75.2% and with a regular occupation in 56.1% of cases.

The mean EAT-26 score was 12.36±10.34. according to this scale, 12.3% of our population were at high risk of developing an ED.

In a multivariate analysis, the female gender (*p* = 0.006), the low economic status (*p* = 0.012), a psychiatric comorbidity (*p* < 0.001), and physical activity (p= 0,037) were strongly associated with ED.

**Conclusions:**

This study highlighted the magnitude of the risk of disordered eating attitudes in the Tunisian population and the need for programs to prevent and control these disorders.

**Disclosure of Interest:**

None Declared

